# Self-Reducible Neglected Recurrent Posterior Hip Dislocation Treated With Cemented Total Hip Arthroplasty

**DOI:** 10.1016/j.artd.2025.101908

**Published:** 2025-11-15

**Authors:** Subhan Shahid, Waqas Ahmad, Abdul Rafeh Awan, Meher Ayyazuddin, Faisal Masood

**Affiliations:** a Department of Orthopaedic Surgery, Unit-II, King Edward Medical University/Mayo Hospital, Lahore, Pakistan; b Department of Surgery, Nishtar Medical University, Multan, Pakistan; c Department of Surgery, CMH Lahore Medical College, Lahore, Pakistan

**Keywords:** Self reduction, Hip dislocation, Neglected dislocation, Total hip arthroplasty, Acetabular fracture

## Abstract

Hip dislocations in adults are uncommon and usually follow high-energy trauma, with neglected cases being rarer but still encountered in low-resource settings. This report describes a 67-year-old man with 6 months of recurrent posterior hip dislocations after an initial injury. He first sought traditional treatment and eventually learned self-reduction techniques. Examination showed limb shortening, muscle wasting, and a neglected posterior acetabular wall fracture with major bone loss. He underwent cemented total hip arthroplasty via a posterior approach, with reconstruction of the posterior wall using an autologous femoral head-neck graft secured by cannulated screws. The case underscores the need for early recognition and proper surgical planning to prevent avascular necrosis, joint degeneration, and long-term disability, especially in developing regions.

## Introduction

Hip dislocations in adults are relatively uncommon and usually result from high-energy trauma. Atraumatic cases are even rarer and often associated with pre-existing abnormalities. Since hip dislocations are considered trauma emergencies, early reduction is critical for favorable outcomes. Consequently, untreated or neglected dislocations are rarely encountered in developed nations with accessible health-care systems [1]. Due to the rarity of such cases, there is limited literature, and no standardized management protocol exists for young adults. Most studies on neglected dislocations involve pediatric patients, typically managed with open reduction, though adult cases pose different challenges [2].

In 1822, Sir Astley Cooper, in A Treatise on Dislocations, and on Fractures of the Joints, described neglected traumatic hip dislocations, categorizing them as upwards (onto dorsum ilii), backwards (into the ischiatic notch), downwards (into the obturator foramen), and anterior (on the pubes). He estimated frequencies as 60% dorsum ilii, 25% ischiatic, 10% obturator, and 5% pubic. These figures align with modern studies, where anterior dislocations (pubic and obturator) represent 7%-13%. Currently, posterior and ischiatic types are generally grouped as posterior [3].

Neglected hip dislocations commonly result from motor vehicle accidents and are often missed when patients have concurrent injuries, such as head trauma or femoral fractures. These associated injuries may draw clinical focus away from the dislocation. Chronic cases are more prevalent in individuals with high pain thresholds, cognitive impairments, or when life-threatening injuries take precedence [4].

In resource-limited settings, patients often seek traditional bone setters, delaying definitive care. Fibrous tissue may fill the acetabulum, complicating reduction, and attempts at delayed reduction risk avascular necrosis (AVN) and arthritis. For dislocations persisting over 3 months, total hip arthroplasty (THA) is advised. Understanding the injury's cause and treatment options is essential, with constrained THA offering a viable approach in chronic or unstable hips [4,5].This paper discusses a rare case of neglected hip dislocation treated with a unique self-reduction technique, ultimately managed successfully with total hip replacement, underscoring THA as a viable solution for neglected or unstable hips.

Informed consent was obtained from the patient for the collection and publication of their clinical data and images.

## Case history

A 67-year-old male patient presented to our outpatient clinic with a 6 month history of recurrent posterior hip dislocations of the right hip. The patient complained of multiple dislocations per day, up to 10 dislocations daily. He reported visiting a local unqualified practitioner for treatment and reduction but to no avail. The patient noted that the dislocations occurred usually during a change of posture, especially while getting up to stand from a sitting position or while rising from the bed. Over time, he developed 2 self-reduction techniques: while standing he applied firm pressure over the greater trochanter to reposition the hip and while supine he dorsiflexed his right foot while applying a downward force with the left foot as shown in Video 1. While these methods initially allowed successful hip reduction, the condition has recently become more painful and difficult to manage due to the increased frequency and discomfort associated with the dislocations.

The patient reported a history of a road traffic accident 9 months earlier, resulting in an unstable posterior fracture-dislocation of the right hip; however, no imagining or documentation was available to confirm this. According to the patient, after an unsuccessful closed reduction attempt, an open reduction and fixation was performed at the hospital. Postoperatively, the patient was non–weight-bearing for 3 months but failed to follow up at the hospital and initiated weight bearing on his own. Shortly after, he developed recurrent posterior dislocations but deferred medical treatment up until now.

**Figure 1:**
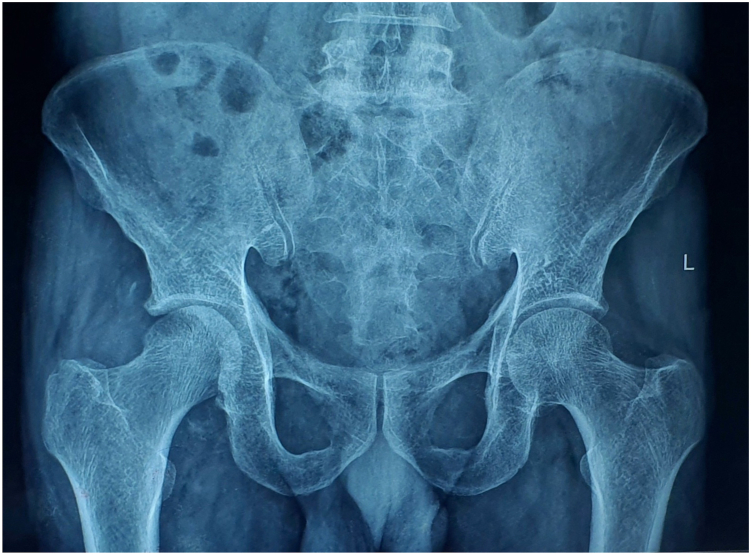
Anteroposterior pelvic radiograph showing right posterior hip dislocation with loss of femoral head congruity and irregularity of the posterior acetabular wall.

**Figure 2:**
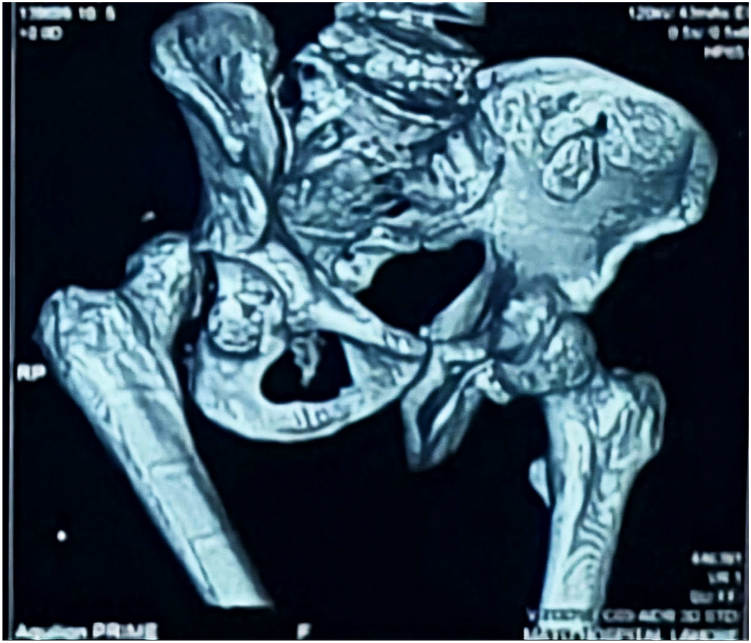
Three-dimensional computed tomography reconstruction demonstrating comminuted fracture of the posterior acetabular wall with substantial bone loss involving more than 50% of the posterior column.

Physical exam revealed a 2.5 cm supratrochanteric shortening with muscle wasting around the hip, thigh and leg. A 15 cm surgical scar was noted, extending over the proximal femur, trochanteric, and gluteal regions. No abductor weakness or neurological deficit was noted in the right lower limb; however, examination for range of motion consistently led to posterior dislocation of the limb. Radiological imaging including an anterior–posterior radiograph and a computed axial tomography scan of the hip highlighted an old, comminuted fracture of the posterior wall of the acetabulum of the right hip with more than half of the wall compromised. (Figs. 1 and 2)

**Figure 3:**
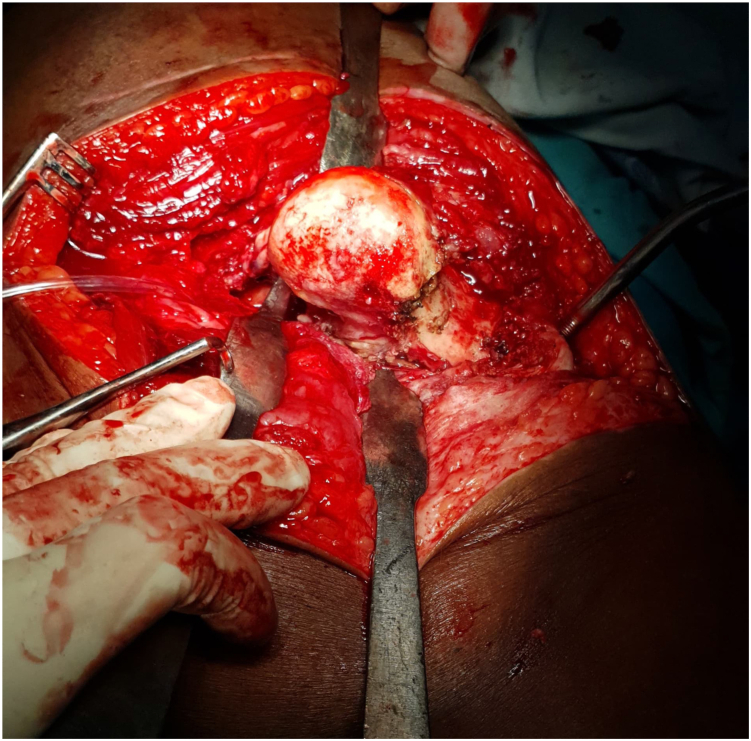
Intraoperative exposure showing the dislocated femoral head after capsulotomy, highlighting scar tissue and fibrous changes due to chronicity.

**Figure 4:**
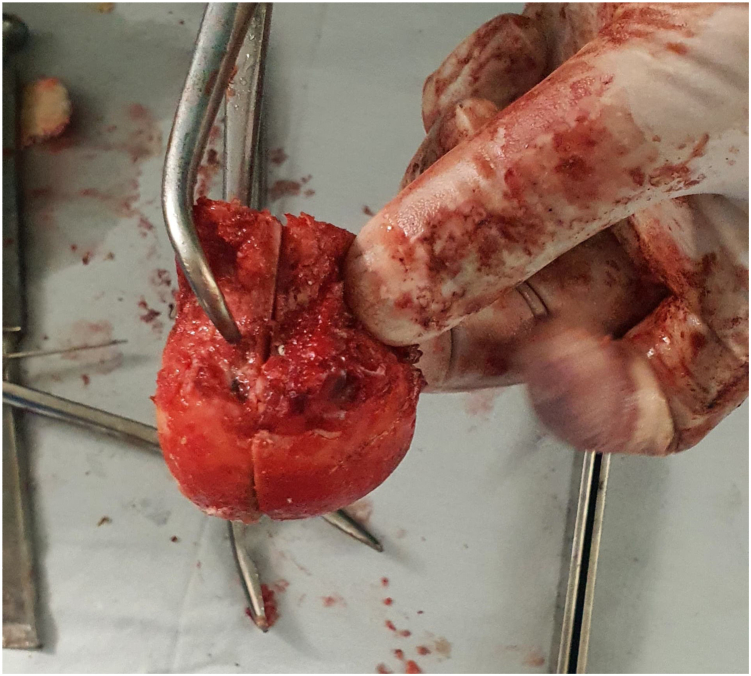
Autologous femoral head graft harvested during femoral neck osteotomy, bisected for posterior acetabular wall reconstruction.

**Figure 5:**
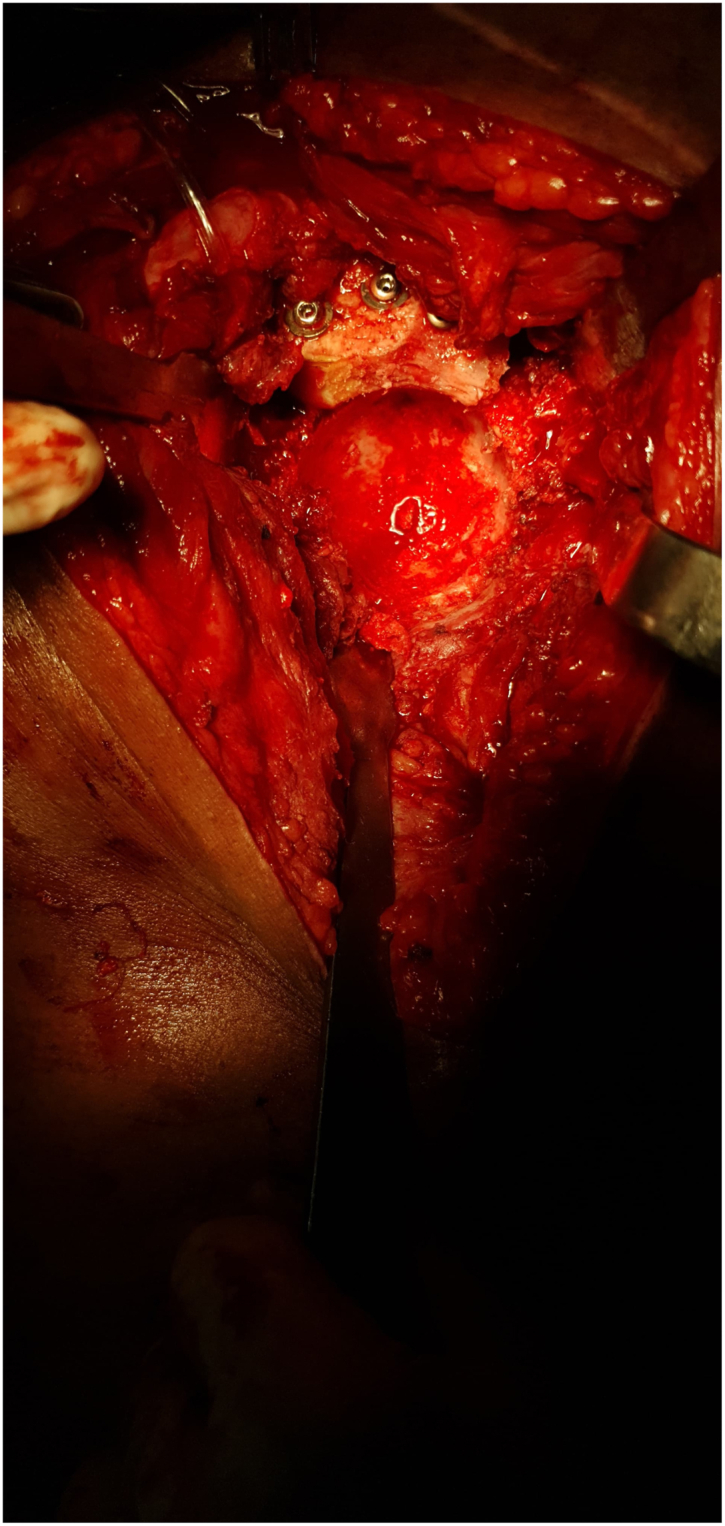
Intraoperative image showing fixation of the femoral head autograft into the posterior acetabular defect using 3 cannulated screws to restore the acetabular wall.

**Figure 6:**
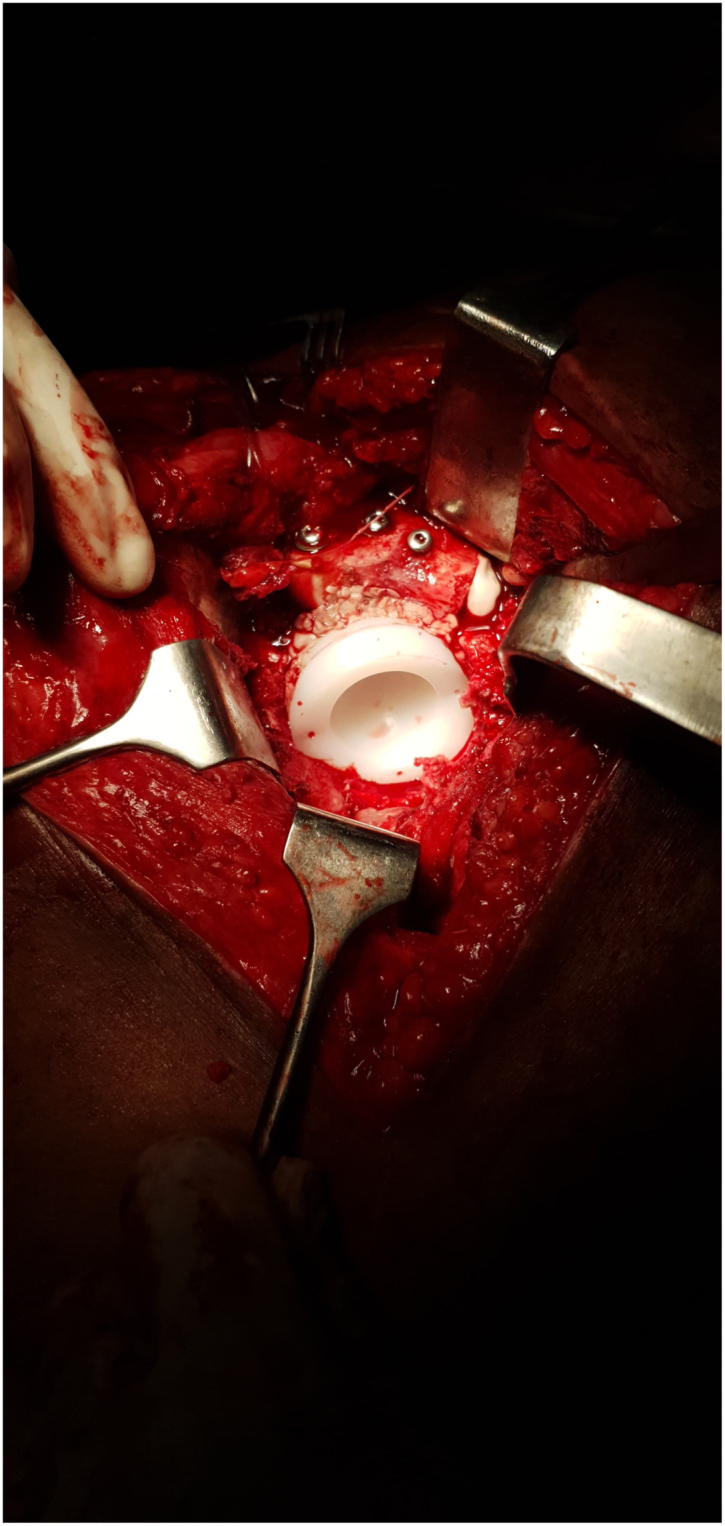
Placement of the cemented acetabular cup following successful reconstruction of the posterior wall with autograft.

**Figure 7:**
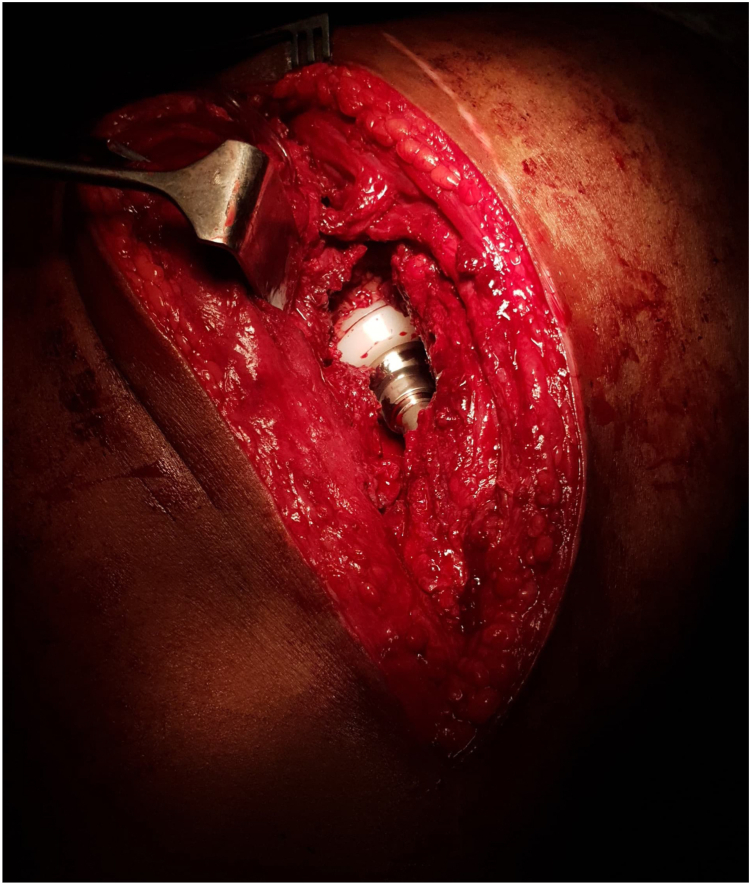
Final intraoperative view showing proper alignment and positioning of the cemented acetabular and femoral components during THA.

**Figure 8:**
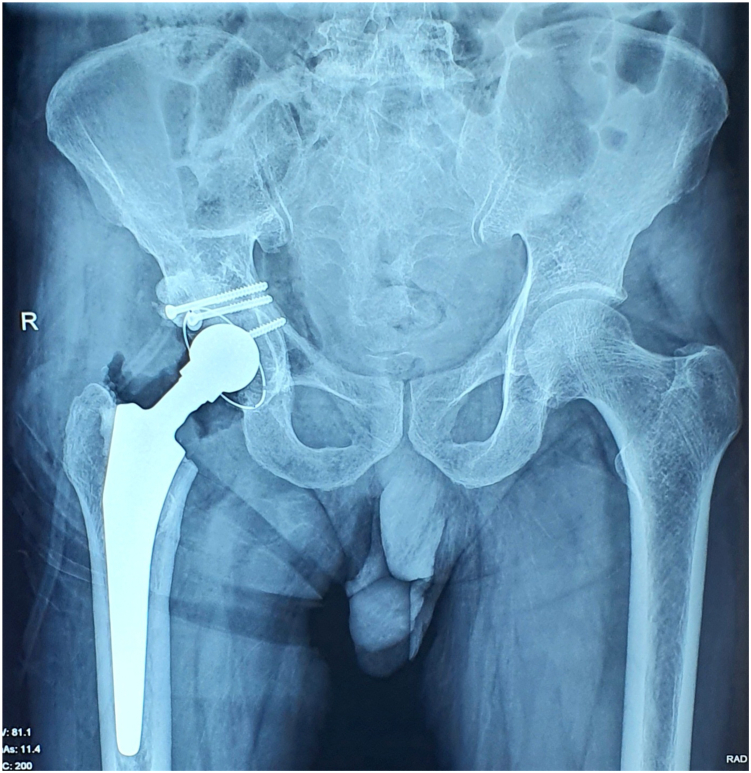
Immediate postoperative radiograph showing well-seated cemented total hip prosthesis with autograft fixation using screws in the reconstructed posterior acetabular wall.

The patient underwent cemented THA using the classical posterior approach [6] (Fig. 3) with reconstruction of the posterior wall defect using autologous graft from the femoral head and neck obtained during osteotomy. The excised femoral head and neck were divided into 2 with 1 portion fashioned to fit the defect in the posterior wall (Fig. 4). The graft was secured with 3 4 mm cancellous screws (32 thread size), measuring 1.8 × 1.8 × 5.5 cm, achieving stable fixation; therefore, additional fixation with a plate was considered unnecessary and abandoned. With the graft successfully restoring the posterior wall integrity, the remaining steps of the cemented hip arthroplasty procedure [7] (Figure 5, Figure 6, Figure 7) proceeded as intended. The combined anteversion achieved was 50°, which was within our intended stability range (Fig. 8). Intraoperatively, hip stability was assessed using multiple maneuvers. The free fall test and shuck test demonstrated satisfactory stability without evidence of subluxation. Additionally, range of motion was evaluated in flexion and adduction without dislocation, and soft-tissue tensioning was performed to confirm appropriate balance prior to closure. Press-fit fixation and jumbo cups were not available in our setting, and due to socioeconomic constraints, a cemented implant with graft reconstruction was selected.

**Figure 9:**
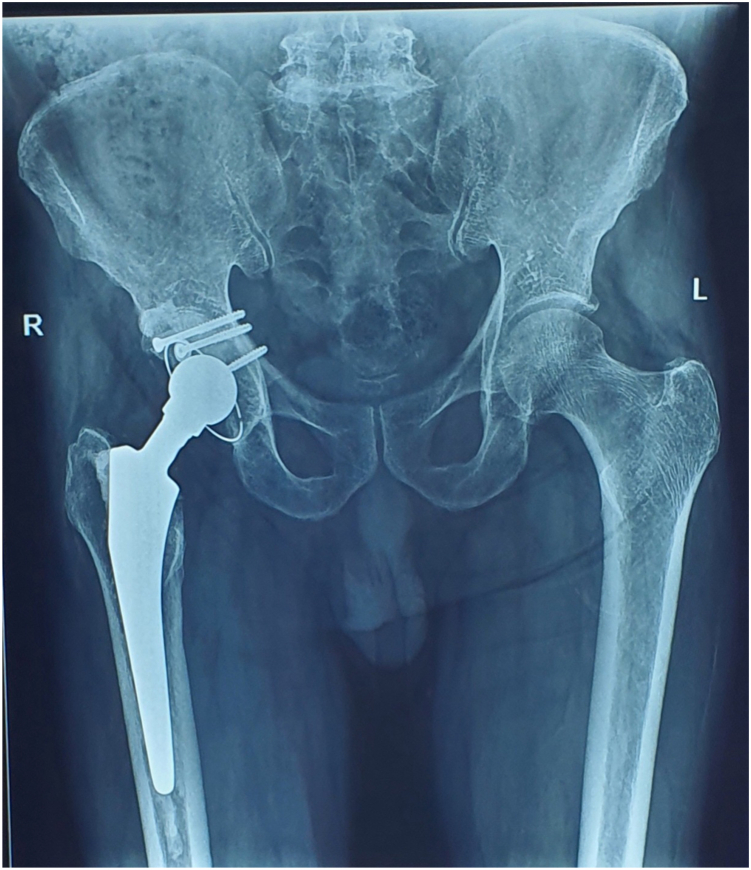
One-year follow-up radiograph showing maintained alignment and integrity of the prosthesis, with no evidence of loosening or dislocation.

The patient was mobilized with toe-touch weight bearing and hip precautions starting on postoperative day 1 and progressed to full weight bearing by 6 weeks. During follow-up visits, there were no episodes of hip dislocation. At the 1-year follow-up, radiographic evaluation (Fig. 9) confirmed a stable reconstruction. The Harris Hip Score improved from 24.2% preoperatively to 91.9% at 1 year, reflecting a marked improvement in functional outcome.

## Discussion

This case highlights the multifaceted challenges of managing neglected hip dislocations, particularly in resource-limited settings, where advanced modular implants and augments were not available. The patient’s unique self-reduction techniques underscore the lengths to which individuals may go when access to qualified medical care is delayed, especially in developing countries where patients delay treatment or prefer treatment by traditional bone setters or osteopaths. These self-reduction practices, while innovative, likely exacerbated the structural damage, contributing to the acetabular defect observed.

Hip dislocations in adults are rare and often result from high-energy trauma, such as falls from height or motor vehicle accidents [8]. Treatment typically involves closed reduction under anesthesia or sedation, with computed tomography scans recommended to assess for associated fractures and injuries; any concurrent fractures, should be managed only after hip reduction. Postreduction, patients are recommended immobilization for 6 weeks followed by partial weight-bearing. Due to the high risk of complications including AVN, fractures, neurovascular damage, and cartilage injury-hip dislocations should be treated as an emergency and require prompt treatment [[8], [9], [10]].

In this case, the delay in treatment was by visiting traditional bone setters leading to severely restricted mobility and prolonged bed confinement. Chronic unreduced hip dislocations carry an almost certain risk of AVN of the femoral head as well as fibrous tissue accumulation in the acetabulum. Prolonged dislocations can also lead to a change in hip biomechanics leading to formation of a pseudoacetabulum and secondary osteoarthritis with severe pain and limited mobility [11].

Treatment options include open reduction, arthrodesis, or THA. Closed reduction is rarely feasible in prolonged dislocations due to soft tissue contractures and acetabular changes. [12]. Even if closed reduction is attempted and complete reduction is achieved, it is often unsuccessful in the long term, especially with coexisting fractures of the acetabulum or femur. As shown by Pai et al. (1992), closed reduction and traction often fail in neglected cases, with most patients ultimately requiring open reduction or reconstruction [13]. This further emphasizes that open reduction is often the necessary approach for neglected hip dislocations with associated fractures.

In open reduction a decision needs to be made between native hip joint preservation or THA. The risk of AVN, chondrolysis, and secondary osteoarthritis in native joints needs to be weighed against the longevity of a prosthesis in THA, especially in younger patients. Hence, the choice of treatment and the procedure itself pose quite a challenge to the surgeon. In the limited literature on neglected hip dislocations, THA is commonly favored by surgeons for neglected hip dislocations of greater than 6 months while others advocate for a 2-stage hip reconstruction using bone grafting [4,14]. This involves a first stage with acetabular reconstruction and a second stage involving arthroplasty. In this case, we used a single-stage reconstruction where we performed an intraoperative acetabular reconstruction followed by arthroplasty. Other options include the use of cement to fill the defect caused by bone loss and arthrodesis, especially in those working as manual labors [14,15].

Delayed acetabular fractures with dislocations pose another significant challenge for surgeons, as the likelihood of achieving accurate reduction diminishes over time. This is often compounded by acetabular fragmentation and head damage which worsen the prognosis. Neglected acetabular fractures are often malunited, complicating reduction and predisposing to secondary arthritis. In such cases, THA with reconstruction remains the most reliable approach [15]. The indications of THA for acetabular fractures include but are not limited to; incomplete fracture reduction, older than 40 years old, prolonged hip dislocation, femoral head articular cartilage injury, acetabular impaction, anterior hip displacement or severe posterior pelvic wall involvement [16].

In elderly patients, where biological healing potential is reduced, it is generally advisable to proceed with single stage reconstruction with bone grafting and arthroplasty [17]. In hip dislocations with fractures of the acetabulum, reconstruction options include bone cement or bone grafts with cemented or cementless implants or reinforcement rings or cages. Gavaskar et al. presented a case like ours, involving a female patient with a 4-month history of an untreated column and posterior wall acetabular fracture, accompanied by a displaced femoral head, which was successfully managed with autografts and cementless THA [18]. Similarly, Pankaj et al. (2011) and Jain et al. (2021) emphasized acetabular reconstruction with grafts to achieve stability. In our case, given the bone morphology, injury pattern, and limitations of a resource-constrained setting, we opted for a cemented THA. Unlike in resource-rich centers where jumbo cups and cementless options may be readily available, public-sector hospitals in developing regions often rely on cemented implants as the only viable option. Despite these limitations, stable fixation and good functional outcomes can still be achieved.

THA generally provides reliable pain relief and functional recovery in neglected hip dislocations, but outcomes are more variable than in primary THA. Case series and reports consistently note higher complication rates, even when long-term function improves [11,19,20]. Metal-on-metal bearings are no longer recommended in younger patients due to concerns of adverse local tissue reactions and high revision risk. Contemporary registry and long-term studies indicate that ceramic-on-ceramic or ceramic-on-highly cross-linked polyethylene bearings offer superior survivorship and wear characteristics, making them the preferred choice in younger, active populations [[21], [22], [23]].

In developing countries, neglected hip dislocations are not uncommon, and patient preferences and expectations should be considered when planning treatment. THA, particularly with advanced bearing options, offers reliable functional recovery [24].

This case is unique in that the patient developed self-reduction techniques to manage recurrent dislocations, and definitive treatment was achieved with autograft reconstruction and cemented THA in a low-resource setting, demonstrating that satisfactory outcomes are possible even when advanced implants are unavailable.

## Summary

This case highlights a rare, self-reduced neglected posterior hip dislocation managed successfully with cemented THA and posterior wall reconstruction using an autologous femoral head graft. It demonstrates the surgical feasibility and favorable outcome of THA in chronic dislocations, emphasizing individualized care in resource-limited or delayed-access scenarios.

## Informed patient consent

The authors confirm that written informed consent has been obtained from the involved patient(s) or if appropriate from the parent, guardian, power of attorney of the involved patient(s); and, they have given approval for this information to be published in this case report (series).

## Conflicts of interest

The authors declare there are no conflicts of interest.

For full disclosure statements refer to https://doi.org/10.1016/j.artd.2025.101908.

## CRediT authorship contribution statement

**Subhan Shahid:** Writing – review & editing, Validation, Supervision, Resources, Investigation, Data curation, Conceptualization. **Waqas Ahmad:** Writing – review & editing, Writing – original draft, Supervision, Investigation, Conceptualization. **Abdul Rafeh Awan:** Writing – review & editing, Writing – original draft, Supervision, Investigation, Conceptualization. **Meher Ayyazuddin:** Writing – review & editing, Writing – original draft. **Faisal Masood:** Writing – review & editing, Supervision, Project administration, Conceptualization.

## Appendix A Supplementary data

Video 1

Haris Hip Score Pre-op

Haris Hip Score at 1 year

Conflict of Interest Statement for Masood

Conflict of Interest Statement for authors Shahid, Ahmad, Awan, and Ayyazuddin
