# Supplementary material for: Self-Reducible Neglected Recurrent Posterior Hip Dislocation Treated With Cemented Total Hip Arthroplasty
Source: Arthroplast Today. 2025 Nov 15;36:101908. doi: 10.1016/j.artd.2025.101908 (PMC12664656; doi:10.1016/j.artd.2025.101908)
Supplement: Haris Hip Score Pre-op [file mmc2.pdf]

orthotoolkit

Harris Hip Score Summary

1. Pain (10 points)
Marked pain, serious limitation of activities

2. Support devices (2 points)
Two canes for support

3. Distance walked (maximum) (2 points)
Walking indoors only

4. Limp (0 points)
Severe limp

5. Put on shoes and socks (2 points)
Puts on shoes and socks with difficulty

6. Stairs (0 points)
Unable to do stairs

7. Enter public transportation (e.g., bus, subway) (0 points)
Unable to use public transportation

8. Sitting (0 points)
Unable to sit comfortably

9. Absence of deformity (4 points)
Patient has no marked physical deformity

10. Total degrees of flexion (3.6 points)
80-90 degrees of flexion

11. Total degrees of abduction (0.4 points)
5-10 degrees of abduction

12. Total degrees of external rotation (0.1 points)
0-5 degrees of external rotation

13. Total degrees of adduction (0.1 points)
5-10 degrees of adduction

Pertinent Negative | Pertinent Positive | Pertinent Positive

Harris Hip Score:
24.20 percent.

Graphical Harris Hip Score

The tools listed on this website do not substitute for the informed opinion of a licensed physician or other health care provider. All scores should be re-checked. Please see our full Terms of Use.
