# Supplementary material for: Self-Reducible Neglected Recurrent Posterior Hip Dislocation Treated With Cemented Total Hip Arthroplasty
Source: Arthroplast Today. 2025 Nov 15;36:101908. doi: 10.1016/j.artd.2025.101908 (PMC12664656; doi:10.1016/j.artd.2025.101908)
Supplement: Haris Hip Score at 1 year [file mmc3.pdf]

orthotoolkit

Harris Hip Score Summary

1. Pain (44 points)
No pain, or ignores it

2. Support devices (11 points)
No support devices necessary

3. Distance walked (maximum) (11 points)
Unlimited walking

4. Limp (11 points)
No limp

5. Put on shoes and socks (4 points)
Puts on shoes and socks with ease

6. Stairs (2 points)
Climbs stairs normally using a railing

7. Enter public transportation (e.g., bus, subway) (1 points)
Able to use public transportation

8. Sitting (3 points)
Able to sit on a high chair for 30 minutes

9. Absence of deformity (0 points)
Patient has physical deformity

10. Total degrees of flexion (3.9 points)
100-110 degrees of flexion

11. Total degrees of abduction (0.6 points)
10-15 degrees of abduction

12. Total degrees of external rotation (0.3 points)
10-15 degrees of external rotation

13. Total degrees of adduction (0.1 points)
5-10 degrees of adduction

Pertinent Negative   Pertinent Positive   Pertinent Positive

Harris Hip Score:
91.90 percent.

Graphical Harris Hip Score

The tools listed on this website do not substitute for the informed opinion of a licensed physician or other health care provider. All scores should be re-checked. Please see our full Terms of Use.
